# Effects of vehicle gap changes on fuel economy and emission performance of the traffic flow in the ACC strategy

**DOI:** 10.1371/journal.pone.0200110

**Published:** 2018-07-12

**Authors:** Xiuhai Li, Tao Yang, Jian Liu, Xiaoqing Qin, Shaowei Yu

**Affiliations:** 1 School of Business, Shandong Yingcai University, Shandong Ji'nan, China; 2 The Joint Laboratory for Internet of Vehicles, Ministry of Education-China Mobile Communications Corporation, Chang'an University, Shaanxi Xi'an, China; Universita degli Studi della Tuscia, ITALY

## Abstract

This paper focuses on the effects of vehicle gap changes on fuel and emission performance of the simulated traffic flow in the adaptive cruise control (ACC) strategy. Firstly, the close correlation of vehicle gap changes and the host car's behaviors was explored with the measured car-following data. Secondly, the correlation between the host car's velocity and vehicle gap changes with different memory steps was also explored to develop the nth car’s optimal velocity function. Thirdly, a microscopic traffic simulation program was created for analyzing the traffic flow evolution process and approximately estimating the fuel consumptions and exhaust emissions. As a result, it was seen that vehicle gap changes with memory significantly affect fuel economy and emission performance of the simulated traffic flow in the ACC strategy, which can result in low fuel consumptions and exhaust emissions. This study is an incremental step forward for designing the control strategy of the ACC system.

## Introduction

With the increasing attention paid to the global energy and environmental issues, quite a lot of projects have been developed. Some focus on reducing size and weight of vehicle bodies, some centre around eco-driving training, and some others are centered on developing some intelligent transportation systems (ITS), which are able to adjust vehicles' speeds based on the preceding vehicles' information [1]. The adaptive cruise control (ACC) system is one of the most favorable ITS, and the existing studies based on microscopic simulations have also indicated that using the ACC system may be a possible solution to improve road traffic efficiency, fuel economy and emission performance of traffic flow [2–17], since the delay time of the controller in them is smaller than the human reaction time [18].

Good properties of traffic flow with the ACC system all rely on high performance control strategies depending on not only the properties of individual vehicles but also their interactions. Lee [19] introduced a memory function into the linear GHR model to store the information of relative speed during CF, which assumes that a driver reacts to the relative speed of the preceding vehicle over a period of time, rather than in an instant value. Zhang [20] developed a continuum macroscopic model arising from a CF model with driver memory, and found that driver memory in CF behaviors can lead to viscous effects in continuum traffic flow dynamics. Sipahi etal [21] analyzed the stability analysis of a constant time-headway driving strategy with driver memory effects modeled by distributed delays. Tang et al. [22] proposed an extended optimal velocity (OV) model with consideration of driver’s memory in the ACC strategy and found that utilizing driver memory to design the ACC strategy can improve the stability of traffic flow. Yu and Shi respectively explored the effects of the velocity changes with memory [23], the relative velocity changes [24,25] and the relative velocity fluctuation [26] on the dynamics and fuel economy of the corresponding traffic flow. Yu and Shi [27] put forward an improved car-following model considering headway changes with memory step of 1s and found that considering headway changes with memory in designing the ACC strategy can improve the stability and fuel economy of traffic flow. Yu and Shi [28] explored the effects of vehicular gap changes with memory on traffic flow in cooperative adaptive cruise control strategy. However, the above studies did not analyze the effects of the vehicle gap with different memory steps on the dynamics, fuel economy and emission performance of the simulated traffic flow in the ACC strategy as well as the nth car’s optimal velocity function. To explore the effects of vehicle gap changes on the dynamics, fuel economy and emission performance of the simulated traffic flow in the ACC strategy as well as the optimal velocity function, this study explores the close relation of vehicle gap changes and the host car's behaviors with the measured data, develop a optimal velocity function and incorporates an improved car-following model considering vehicle gap changes[28] to capture the operations of the ACC traffic flow system and then utilizes the VT-Micro model to estimate fuel consumption and exhaust emissions.

## Data collection and mining analysis

Here, we select the signalized intersection of Jingshi Road/Shanshi East Road of Jinan in China. This intersection is located in the downtown area and on the major arterial, and it can completely meet the needs of real-time data acquisition.

We only record and analyze the movements of the CF vehicles on the middle three through lanes. The recording time is from 2:00 PM to 5:00 PM on December 11, 2013. The CF data in seconds are extracted by using the frame differential method, the raw data are preprocessed utilizing a linear transformations technique. The measured CF data are obtained and some are listed as shown in S1, S2 and S3 Tables.

The gray correlation degree [29,30] is a quantitative value of the correlation between the behavior factors. Higher is the value of the gray correlation degree, more relevant are the main-factor and sub-factor. The interacting car-following process can be regarded as a nonlinear stochastic system. The paper uses the gray correlation analysis theory to test whether or not vehicle gap changes with memory greatly affect the following car’s behaviors as well as the nth car’s optimal velocity function. The corresponding gray correlation degrees are obtained and listed as shown in Tables 1 and 2.

**Table 1:** Results of gray correlation analysis.

Time steps	d_21_	v_1_	Δv_21_	Δx_m21_
**δ = 1**	0.4466	0.5227	0.9125	0.9176
**δ = 2**	0.5017	0.5760	0.9288	0.8675
**δ = 3**	0.6439	0.7137	0.9612	0.8794

where δ is the memory time step.

**Table 2:** Results of gray correlation analysis.

Time steps	δ = 1s	δ = 2s	δ = 3s
**d**_**21**_	0.9595	0.9161	0.9616
**Δx**_**m21**_	0.8741	0.8642	0.8845

In Table 1, it can be obviously found that Δv_*21*_ and Δx_*m21*_ are more similar with a_*1*_ than d_*21*_ and v_*1*_, and the similarities of Δv_*21*_ and Δx_*m21*_ are much the same, and that Δx_*m21*_ have significant effects on the host car's behaviors.

In Table 2, it can be obviously found that d_21_ is more similar with v_1_ than Δx_m21,_ and the similarities of d_21_ and Δx_m21_ are all greater than 0.86, and that d_21_ and Δx_m21_ are highly relevant to the following car's velocity.

## The related models

### The CF model considering vehicle gap changes

Yu and Shi [27] put forward an improved car-following model considering headway changes with memory step of 1s and found that considering headway changes with memory in designing the ACC strategy can improve the stability and fuel economy of traffic flow. However, the above study did not analyze the effects of the vehicle gap with different memory steps on the dynamics, fuel economy and emission performance of the simulated traffic flow in the ACC strategy as well as the nth car’s optimal velocity function. Thus, we develop the optimal velocity function and incorporate the improved CF model considering vehicle gap changes to capture the operations of the ACC traffic flow system. And the improved CF model is expressed as

$${\ddot{x}}_{n}\left(t\right)=\kappa \left[V\left(\Delta x_{n}\left(t\right)\right)-v_{n}\left(t\right)\right]+\lambda \Delta v_{n}\left(t\right)+\gamma \left[\Delta x_{n}\left(t\right)‑\Delta x_{n}\left(t‑\delta \right)\right]$$ (1)

where *x_n_*(*t*) is the position of car *n* at the time *t*; *V*(.) is the optimal velocity function; Δ*x_n_*(*t*) and Δ*v_n_*(*t*) are the relative distance and the relative velocity between car *n* and car *n+1* at the time *t*; [Δ*x*_*n*+1_(*t*)-Δ*x*_*n*+1_(*t*-*δ*)] is vehicle gap changes with different time steps; *κ*, *λ* and *γ* are respectively sensitivity parameters.

Based on the above results of Table 2, it can be obviously found that d_21_ and Δx_m21_ are highly relevant to the following car's velocity. Therefore, the *n*th car’s optimal velocity function can be developed as follows:

$$V\left(\Delta x_{n}\left(t\right)\right)=V_{1}+V_{2}\tanh(C_{1}(\Delta x_{n}\left(t\right)-l)-C_{2})+V_{3}[\Delta x_{n}\left(t\right)-\Delta x_{n}\left(t-\delta \right)]$$ (2)

where V_1_, V_2_, V_3_, C_1_ and C_2_ are respectively sensitivity parameters, *l* is the car's length.

The proposed model can be reduced to the full velocity difference model when δ = 0s.

### The VT-Micro model

Ahn [31] applied the data transformation technique to develop a VT-Micro model. The VT-Micro model was proposed as a statistical model consisting of linear, quadratic and cubic combinations of speed and acceleration levels using the measured data collected at the Oak Ridge National Laboratory and the Environmental Protection Agency. The calibrated model can provided a perfect fit for all measures of effectiveness tested, which can be expressed as:

$$\ln(MOE_{e})=\sum_{i=0}^{3}\sum_{j=0}^{3}\left(K_{i,j}^{e}\times v^{i}\times {\left(\frac{dv}{dt}\right)}^{j}\right)$$ (3)

where MOE_e_ is car’s instantaneous fuel consumption rate or exhaust emission rate, $K_{i,j}^{e}$ is the model regression coefficient for MOE “e” at speed power “i” and acceleration power “j” for negative accelerations, *v* is the instantaneous speed(m/s), *dv*/*dt* is the instantaneous acceleration(m/s^2^).

## Simulation analysis of the traffic flow evolution

In this section, numerical simulations under the periodic boundary condition are carried out to analyze the traffic flow evolution process in the ACC strategy influenced by vehicle gap changes with different memory steps. The initial conditions are set as follows: 70 cars are distributed on the ring road with the length L = 1050m uniformly. The initial disturbance is supposed that the initial gap between car 69 and car 70 is 5 m, the initial gap between car 69 and car 68 is 15 m, and the others are 10 m. The memory steps are respectively set as 0s, 1s, 2s, 3s and 4s for comparative analysis, *γ* = 0.1, the parameters of the optimal velocity function are obtained by calibrating with the measured data, and the other parameters are adopted as same as those in the research study [7].

**Fig 1:**
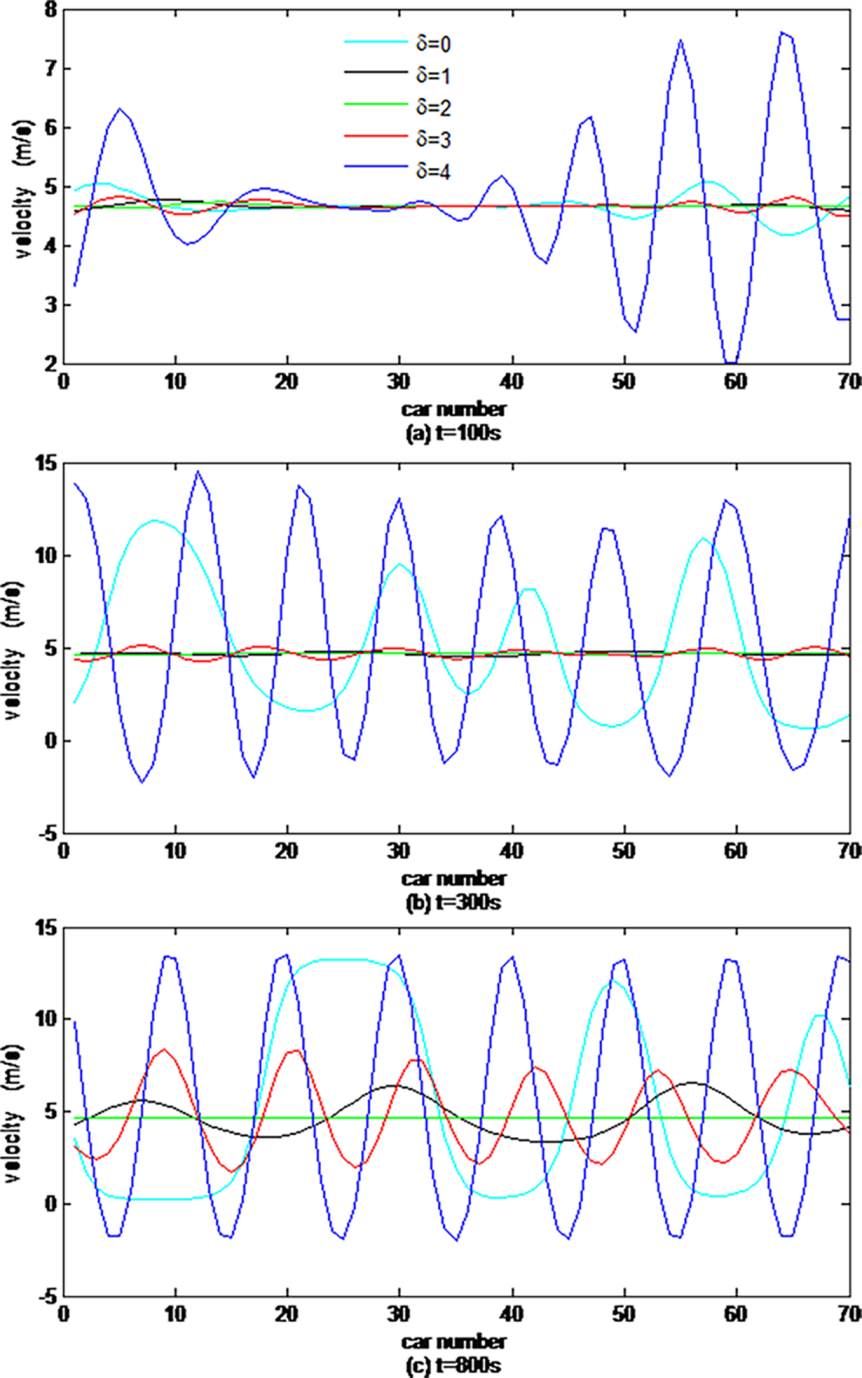
Velocity distributions of 70 cars simulated by the CF models with different memory steps.

First, we explore the effects of vehicle gap changes with different memory steps in the ACC strategy on the velocity evolution process. Fig 1 illustrates velocity distributions obtained at the time steps of t = 100s, 300s and800s, where the different curves stand for velocity distributions of 70 cars simulated by the CF model, which consider vehicle gap changes with different memory steps.

From Fig 1, it can be obviously found that the velocities of all vehicles fluctuate around the initial velocity v0 = 4.6647m/s between the minimum and maximum caused by the initial small disturbance, however, the difference between them cannot be intuitively distinguished.

To distinguish the distinction caused by vehicle gap changes with different memory steps in the ACC strategy explicitly, the standard deviations of velocity distribution are obtained and listed in Table 3.

**Table 3:** The standard deviation of velocity distribution.

Time	δ = 0s	δ = 1s	δ = 2s	δ = 3s	δ = 4s
**t = 50s**	0.0838	0.0335	0.0275	0.0592	0.2236
**t = 100s**	0.1900	0.0364	0.0213	0.0720	1.1893
**t = 200s**	1.1146	0.0525	0.0165	0.1243	5.0470
**t = 300s**	3.5854	0.0814	0.0143	0.2300	5.4468
**t = 500s**	4.9357	0.2114	0.0119	0.7966	5.6530
**t = 800s**	4.9681	0.9770	0.0101	2.0353	5.6792
**t = 1000s**	5.4117	2.4033	0.0094	2.3837	5.6806
**t = 2000s**	5.5313	3.8565	0.0075	2.6272	5.6807
**t = 3000s**	5.5312	3.8565	0.0066	2.2357	5.6807

From Table 3, it can be obviously found that the velocities of all vehicles fluctuate around the initial velocity caused by the initial small disturbance, that the standard deviations of velocity distribution first gradually descend, and then ascend with the increase of the memory steps, and that the range of all vehicles' velocity fluctuation is smallest when the memory step is 2s.

**Fig 2:**
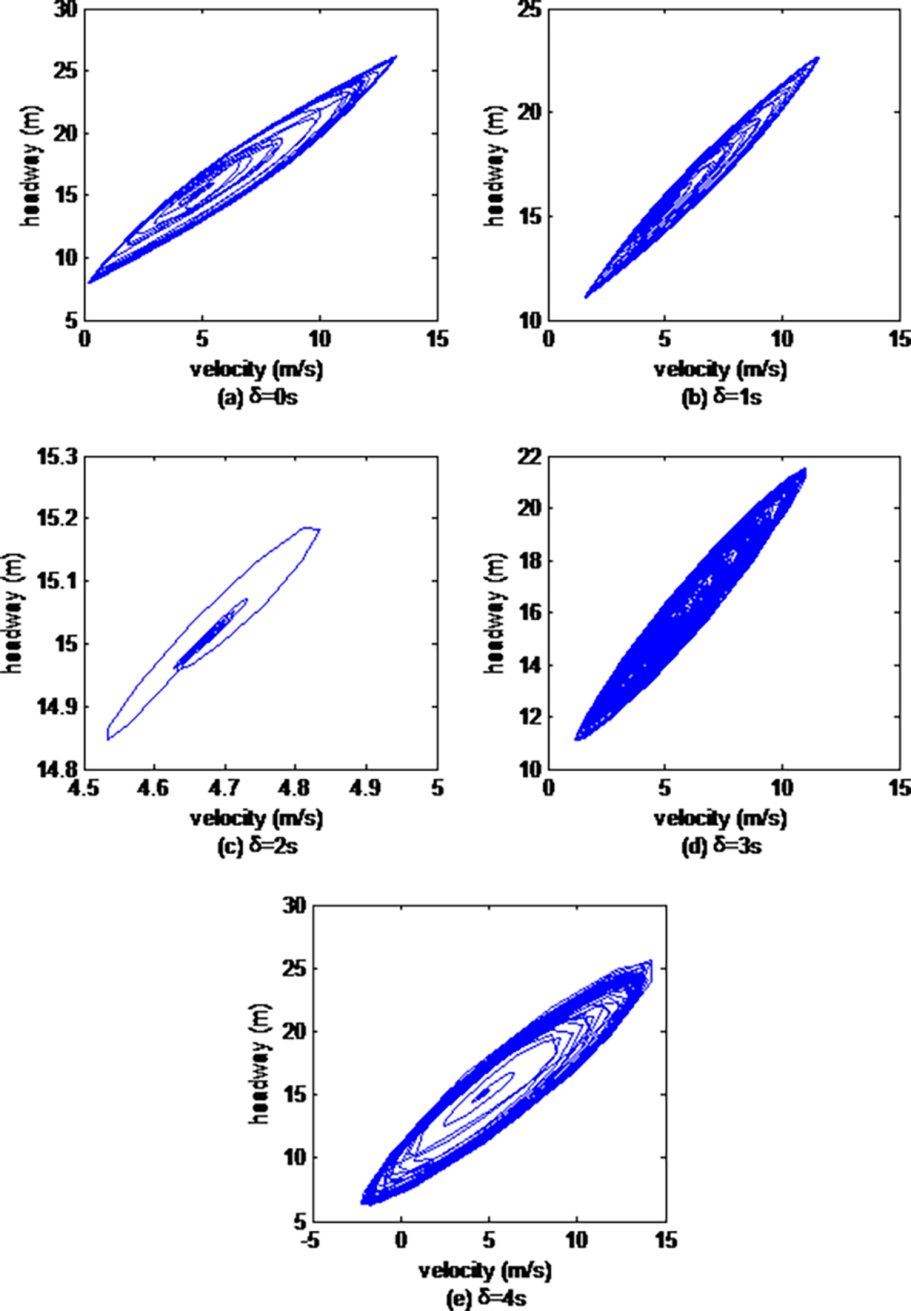
Hysteresis loops from the 10th car simulated by CF model.

**Fig 3:**
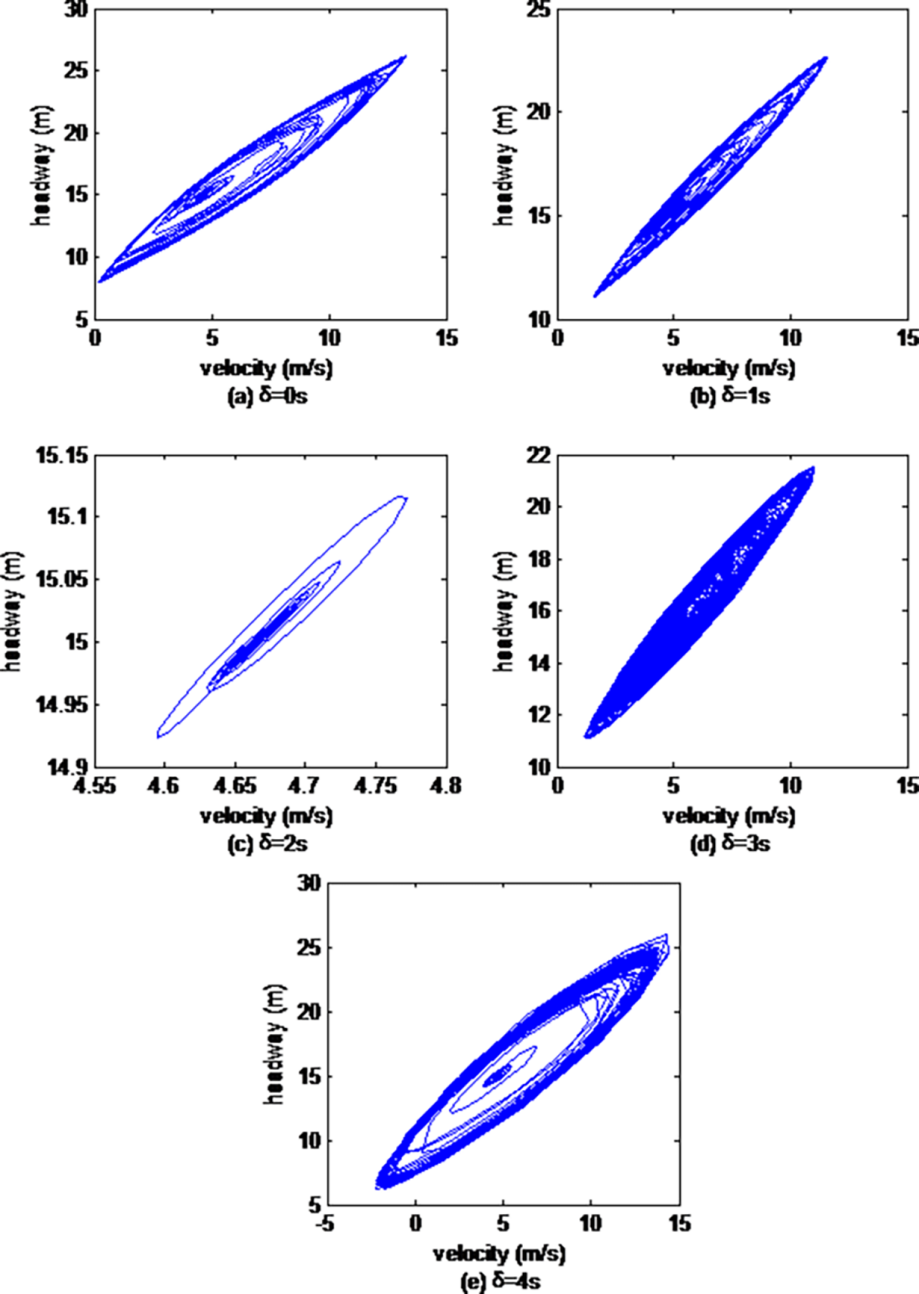
Hysteresis loops from the 30th car simulated by CF model.

**Fig 4:**
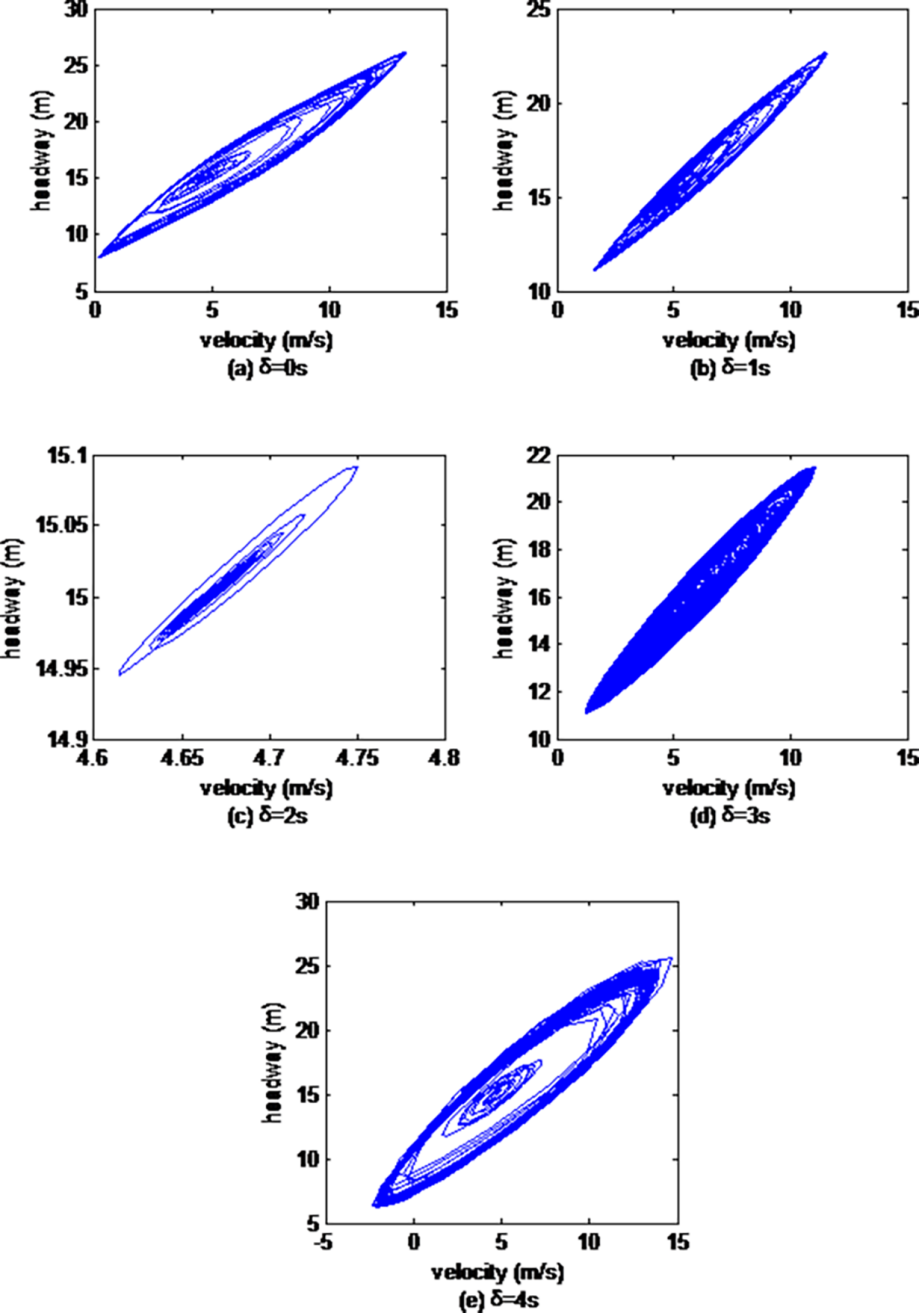
Hysteresis loops from the 50th car simulated by CF model.

Then, we explore the impacts of vehicle gap changes with different memory steps in the ACC strategy on the headway evolution process. Figs 2, 3 and 4 illustrate hysteresis loops simulated by the 10th, the 30th and the 50th cars respectively.

As can be seen from Figs 2, 3 and 4, the sizes of the hysteresis loops all first gradually descend, and then rise with the increase of the memory steps, and the size of hysteresis loops is smallest when the memory step is 2s, which is in accordance with the results of the above velocity evolution analysis.

The analysis of the above stop-and-go charts and hysteresis loops prove that vehicle gap changes with different memory steps have significant and different effects, that the stability of the traffic flow is optimal when the memory step is 2s.

## Fuel economy and emissions estimation

Vehicles ' driving behaviors have been seen to offer considerable potential methods for reducing fuel consumptions and exhaust emissions [32–36]. Whether considering vehicle gap changes with different memory steps in design of the ACC strategy can affect fuel economy and emission performance of the simulated traffic flow need to be further investigated. The VT-Micro model is employed to explore the impacts of vehicle gap changes with different memory steps on the fuel consumptions and emission performance on the basis of the above numerical simulations under the periodic boundary condition.

First, we study the impacts of vehicle gap changes with different memory steps in the ACC strategy on the each car’s instantaneous fuel consumption and the total fuel consumptions of the whole CF system. The parameters of the VT-micro model can be obtained from the literature [34] as shown in the S4 Table.

**Fig 5:**
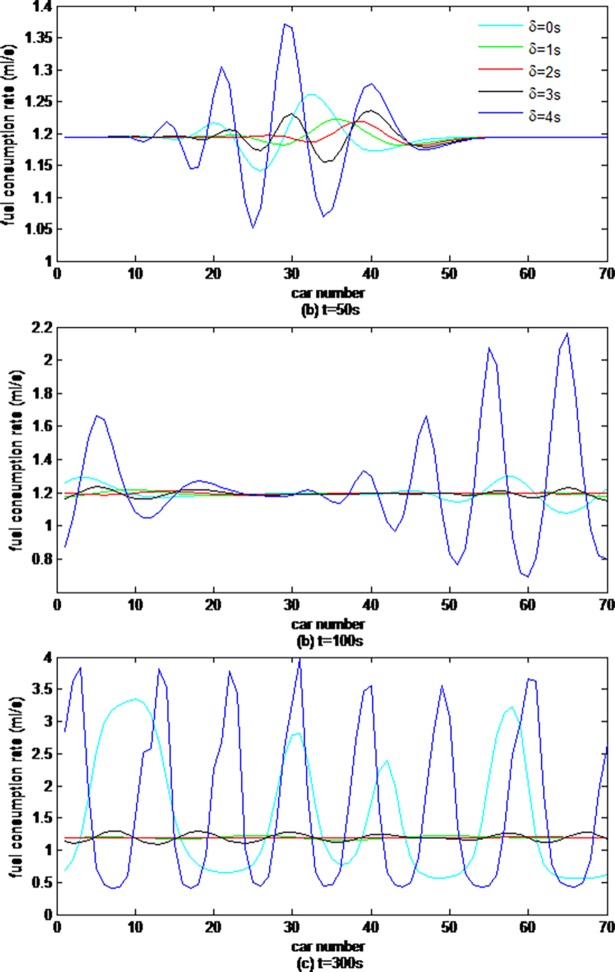
The fuel consumption rate of 70 cars simulated by different CF models.

Fig 5 depicts the fuel consumption rate of each car simulated by the CF model considering vehicle gap changes with different memory steps at the time steps of t = 50s, 100s and 300s.

From Fig 5, it can be found that all vehicles' instantaneous fuel consumption fluctuate around the initial value between the minimum and maximum caused by the initial disturbance, that the fluctuation range of the instantaneous fuel consumption fluctuation gradually descends firstly, and then ascends, and that the fluctuation range is smallest when the memory step is 2s, which is in accordance with the results of the above traffic flow evolution process.

Table 4 lists the total fuel consumptions of the whole CF system respectively simulated by the CF model considering vehicle gap changes with different memory steps during different period.

**Table 4:** The total fuel consumption of the whole CF system.

time	δ = 0s	δ = 1s	δ = 2s	δ = 3s	δ = 4s
**t = 50s**	4182. 5	4182.13	4182.12	4182.34	4183.85
**t = 100s**	8366.06	8363.60	8363.51	8364.16	8414.2
**t = 200s**	16814.0	16726.7	16726.3	16729.0	18843.5
**t = 300s**	26357.1	25090.4	25089.0	25098.7	30488.0
**t = 500s**	48235.3	41825.1	41814.3	41935.2	54178.9
**t = 800s**	81416.6	67118.8	66902.4	68575.6	89822.9
**t = 1000s**	103236	85087.4	83627.7	87371	113593
**t = 2000s**	213871	187626	167254	182893	232444
**t = 3000s**	324499	291853	250881	276045.6	351294.7

As can be seen from Table 4, the total fuel consumptions of the whole CF system first gradually descends, and then rises with the increase of the memory steps, and that the fuel economy of the traffic flow is optimal when the memory step is 2s.

Next, we implement further study on the impacts of vehicle gap changes with different memory steps on exhaust emissions. Tables 5, 6 and 7 respectively list the total CO, HC and NO_X_ emissions of the whole CF system.

**Table 5:** The total CO emissions of the whole CF system.

time	δ = 0s	δ = 1s	δ = 2s	δ = 3s	δ = 4s
**t = 50s**	28880.2	28872.6	28872.3	28876.8	28907.3
**t = 100s**	57784.5	57735.2	57733.4	57746.5	58838.5
**t = 200s**	117330	115464	115454	115510	217360
**t = 300s**	217812	173205	173175	173373	505371
**t = 500s**	672301	288835	288615	291120	1143110
**t = 800s**	1503243	466396	461775	500251	2129430
**t = 1000s**	2078864	616127	577214	672007	2788909
**t = 2000s**	5466525	2176317	1154410	1707450	6086978
**t = 3000s**	8858945	3940903	1731607	2701972	9385067

**Table 6:** The total HC emissions of the whole CF system.

time	δ = 0s	δ = 1s	δ = 2s	δ = 3s	δ = 4s
**t = 50s**	2948.31	2947. 80	2947.78	2948.08	2950.15
**t = 100s**	5898.24	5894.92	5894.80	5895.69	5970.8
**t = 200s**	11916.85	11789	11788.8	11793	23546
**t = 300s**	21503.82	17685	17682.7	17696	65236
**t = 500s**	75906.65	29485	29471	29640	158635
**t = 800s**	187013	47465	47152	49832	304796
**t = 1000s**	266891	61749	58940	65765	402578
**t = 2000s**	765711	216273	117879	162772	891599
**t = 3000s**	1265854	395536	176817	257630	1380627

**Table 7:** The total NO_X_ emissions of the whole CF system.

time	δ = 0s	δ = 1s	δ = 2s	δ = 3s	δ = 4s
**t = 50s**	4342.59	4341.962	4341.955	4342.37	4345.17
**t = 100s**	8686.88	8682.77	8682.64	8683.81	8778.59
**t = 200s**	17512.1	17364.7	17363.9	17368.8	22814.6
**t = 300s**	28461	26048	26045	26062	41915
**t = 500s**	57302	43425	43408	43621	82849
**t = 800s**	102533	69808.	69451	72502	145129
**t = 1000s**	132264	89361	86814	93852	186721
**t = 2000s**	287482	214985	173625	205007	394709
**t = 3000s**	442729	345332	260437	311763	602698

From Tables 5, 6 and 7, it can be obviously found that the total CO, HC and NO_X_ emissions of the whole CF system all gradually descend firstly, and then ascend with the increase of the memory steps during different period of time steps, which is in accordance with the results of the above fuel economy analysis.

### Conclusions

The data mining analysis shows that vehicle gap changes with different memory steps have obviously different effects on the host car's behaviors. The analysis results of several numerical simulations indicate that considering vehicle gap changes with different memory steps in designing the control strategy for the ACC system can improve the stability and fuel economy, as well as emissions performance of the simulated traffic flow. However, there are some limitations in this paper as follows:

1. We only obtain the measured CF data with three memory steps for the time being, due to the limitation of visual angle.
2. The communication delay and the time delay in the controller of the ACC system are not considered explicitly for the present.

In the further work, the image fusion technologies are used to collect the measured CF data with more memory steps, the communication delay and the time delay in the controller are considered to develop an effective CF model to study the effects on the dynamic, fuel economy and exhaust emission performance of the corresponding traffic flow by analytical analysis and numerical simulations.

## Supporting information

S1 Table Partial measured CF data (δ = 1s). (DOC)

S2 Table Partial measured CF data (δ = 2s). (DOC)

S3 Table Partial measured CF data (δ = 3s). (DOC)

S4 Table The related coefficients in Eq (6). (DOC)
