# Supplementary material for: Effects of vehicle gap changes on fuel economy and emission performance of the traffic flow in the ACC strategy
Source: PLoS One. 2018 Jul 12;13(7):e0200110. doi: 10.1371/journal.pone.0200110 (PMC6042720; doi:10.1371/journal.pone.0200110)
Supplement: S1 Table — (DOC) [file pone.0200110.s001.doc]

**S1 Table. Partial measured CF data (δ=1s**)

| **t** | **a_1_(m/s^2^)** | **d_21_(m)** | **v_1_(m/s)** | **Δv_21_(m/s)** | **Δxc_21_(m/s)** |
| --- | --- | --- | --- | --- | --- |
| 1 | -0.6665 | 4.73215 | 4.99875 | -0.6665 | -0.46655 |
| 2 | -0.6665 | 4.33225 | 4.33225 | -0.6665 | -0.59985 |
| 3 | -0.6665 | 3.93235 | 3.66575 | -0.6665 | -0.86645 |
| 4 | -0.6665 | 3.3325 | 2.99925 | -0.6665 | -1.26635 |
| 5 | -0.6665 | 2.86595 | 2.33275 | -0.6665 | -1.36633 |
| 1 | -0.6665 | 8.63118 | 4.99875 | -0.99975 | -0.46655 |
| 2 | -0.6665 | 7.39815 | 4.33225 | -1.333 | -0.43323 |
| 3 | -1.333 | 6.1318 | 3.3325 | -1.333 | -0.73315 |
| 1 | -0.6665 | 9.76423 | 3.66575 | -0.99975 | -0.63318 |
| 2 | -0.6665 | 8.1313 | 2.99925 | -1.333 | -0.19995 |
| 3 | -0.6665 | 6.2651 | 2.33275 | -1.333 | 0.1333 |
| 1 | -0.6665 | 8.83113 | 4.33225 | -0.99975 | -0.49988 |
| 2 | -0.6665 | 7.69808 | 3.66575 | -0.99975 | -0.59985 |
| 3 | -0.6665 | 6.56503 | 2.99925 | -1.333 | -0.43323 |
| 1 | -0.6665 | 8.7978 | 5.66525 | -0.6665 | -0.6665 |
| 2 | 0 | 7.69808 | 5.332 | -0.99975 | -1.29968 |
| 3 | -0.6665 | 6.33175 | 4.99875 | -1.9995 | -2.1328 |
| 1 | 0.6665 | 4.9321 | 4.99875 | -1.9995 | -1.13305 |
| 2 | -2.666 | 3.13255 | 3.999 | -1.9995 | -1.16638 |
| 3 | -1.333 | 1.93285 | 1.9995 | -0.6665 | -0.9331 |
| 1 | -0.6665 | 2.5327 | 4.33225 | -0.6665 | -0.29993 |
| 2 | -1.333 | 1.56628 | 3.3325 | -0.99975 | -0.76648 |
| 3 | -1.333 | 1.26635 | 1.9995 | -0.6665 | -0.96643 |
| **1** | 0 | 5.098725 | 4.6655 | 0.99975 | 0.299925 |
| **2** | -0.6665 | 5.098725 | 4.33225 | 0.33325 | 0 |
| **3** | -0.6665 | 4.86545 | 3.66575 | -0.6665 | -0.233275 |
| **4** | -1.333 | 4.33225 | 2.666 | -0.6665 | -0.5332 |
| **1** | -0.6665 | 4.2656 | 5.66525 | 0.6665 | 0.6665 |
| **2** | -0.6665 | 4.698825 | 4.99875 | 0.33325 | 0.433225 |
| **3** | -0.6665 | 4.33225 | 4.33225 | -0.6665 | -0.366575 |
| **4** | -1.333 | 3.699075 | 3.3325 | -0.33325 | -0.633175 |
| **1** | -0.6665 | 16.029325 | 4.99875 | -2.33275 | -1.93285 |
| **2** | -0.6665 | 14.096475 | 4.33225 | -1.66625 | -1.93285 |
| **3** | -0.6665 | 12.096975 | 3.66575 | -1.66625 | -1.9995 |
| **4** | -0.6665 | 9.9975 | 2.99925 | -1.9995 | -2.099475 |
| **1** | -0.6665 | 6.1318 | 4.99875 | 0 | -0.19995 |
| **2** | -0.6665 | 5.8652 | 4.33225 | 0 | -0.2666 |
| **3** | -0.6665 | 5.431975 | 3.66575 | -0.33325 | -0.433225 |
| **4** | -0.6665 | 4.565525 | 2.99925 | -0.99975 | -0.86645 |
| **1** | 0 | 6.431725 | 5.332 | -0.33325 | 0.06665 |
| **2** | -0.6665 | 6.19845 | 4.99875 | -0.6665 | -0.233275 |
| **3** | -0.6665 | 5.93185 | 4.33225 | -0.6665 | -0.2666 |
| **4** | -0.6665 | 5.53195 | 3.66575 | -0.6665 | -0.3999 |
| **5** | -0.6665 | 4.99875 | 2.99925 | -0.6665 | -0.5332 |
| **1** | -0.6665 | 7.364825 | 4.99875 | -0.99975 | -0.633175 |
| **2** | -0.6665 | 6.7983 | 4.33225 | -0.6665 | -0.566525 |
| **3** | -0.6665 | 5.93185 | 3.66575 | -0.6665 | -0.86645 |
| **4** | -0.6665 | 5.165375 | 2.99925 | -0.33325 | -0.766475 |
| **1** | 0 | 3.965675 | 3.999 | -0.33325 | -0.233275 |
| **2** | -1.333 | 3.632425 | 3.3325 | -0.33325 | -0.33325 |
| **3** | 0 | 3.4658 | 2.666 | 0 | -0.166625 |
| **4** | -0.6665 | 3.165875 | 2.33275 | -0.33325 | -0.299925 |
| **5** | -0.6665 | 2.699325 | 1.66625 | -0.33325 | -0.46655 |
| **1** | -0.6665 | 6.9316 | 6.33175 | -1.333 | -0.73315 |
| **2** | -1.333 | 6.59835 | 5.332 | -0.6665 | -0.33325 |
| **3** | -0.6665 | 6.2651 | 4.33225 | 0 | -0.33325 |
| **4** | 0 | 5.7319 | 3.999 | -0.6665 | -0.5332 |
| **1** | -1.333 | 6.764975 | 6.665 | -1.66625 | -1.832875 |
| **2** | -1.333 | 5.032075 | 5.332 | -0.99975 | -1.7329 |
| **3** | -0.6665 | 3.79905 | 4.33225 | -0.6665 | -1.233025 |
| **4** | -0.6665 | 3.232525 | 3.66575 | -0.99975 | -0.566525 |
| **1** | -0.6665 | 5.26535 | 5.66525 | -0.6665 | -0.433225 |
| **2** | -1.333 | 4.59885 | 4.6655 | -0.6665 | -0.6665 |
| **3** | -0.6665 | 3.999 | 3.66575 | 0 | -0.59985 |
| **4** | 0 | 3.765725 | 3.3325 | -0.33325 | -0.233275 |
| **5** | -0.6665 | 2.9326 | 2.99925 | -1.333 | -0.833125 |
| **1** | 0 | 4.432225 | 5.332 | 0.33325 | 0.233275 |
| **2** | -0.6665 | 4.232275 | 4.99875 | -0.33325 | -0.19995 |
| **3** | -1.333 | 3.5991 | 3.999 | -0.6665 | -0.633175 |
| **4** | -0.6665 | 2.99925 | 2.99925 | -0.6665 | -0.59985 |
| **1** | -1.333 | 12.563525 | 5.9985 | -0.99975 | -0.899775 |
| **2** | -0.6665 | 11.5971 | 4.99875 | -0.6665 | -0.966425 |
| **3** | 0 | 10.364075 | 4.6655 | -0.99975 | -1.233025 |
| **4** | -0.6665 | 9.097725 | 4.33225 | -1.66625 | -1.26635 |
| **1** | -0.6665 | 6.2651 | 4.99875 | 0.6665 | 0.299925 |
| **2** | 0 | 6.365075 | 4.6655 | 0.33325 | 0.099975 |
| **3** | -1.333 | 5.8652 | 3.999 | 0 | -0.499875 |
| **4** | 0 | 4.965425 | 3.3325 | -0.33325 | -0.899775 |
| **5** | -0.6665 | 4.06565 | 2.99925 | -1.333 | -0.899775 |
| **1** | -0.6665 | 8.497875 | 4.33225 | -0.33325 | 0.033325 |
| **2** | -0.6665 | 8.33125 | 3.66575 | -0.6665 | -0.166625 |
| **3** | -0.6665 | 7.4648 | 2.99925 | -0.6665 | -0.86645 |
| **4** | -0.6665 | 6.19845 | 2.33275 | -0.6665 | -1.26635 |
| **1** | -0.6665 | 10.697325 | 5.66525 | -0.99975 | -0.99975 |
| **2** | -0.6665 | 9.1977 | 4.99875 | -1.333 | -1.499625 |
| **3** | -1.333 | 7.698075 | 3.999 | -0.99975 | -1.499625 |
| **4** | -1.333 | 6.33175 | 2.666 | -0.6665 | -1.366325 |
| **1** | -0.6665 | 5.79855 | 4.33225 | 0 | -0.499875 |
| **2** | 0 | 5.0654 | 3.999 | -0.6665 | -0.73315 |
| **3** | -1.333 | 4.098975 | 3.3325 | -0.99975 | -0.966425 |
| **4** | -0.6665 | 3.26585 | 2.33275 | -0.6665 | -0.833125 |
| **5** | -0.6665 | 2.46605 | 1.66625 | -0.6665 | -0.7998 |
| **1** | -0.6665 | 2.19945 | 3.66575 | 0 | -0.2666 |
| **2** | -0.6665 | 2.1328 | 2.99925 | 0.6665 | -0.06665 |
| **3** | 0 | 2.232775 | 2.666 | 0.33325 | 0.099975 |
| **4** | -1.333 | 2.032825 | 1.9995 | -0.33325 | -0.19995 |
| **1** | -0.6665 | 11.163875 | 4.99875 | -0.33325 | -0.5332 |
| **2** | 0 | 10.630675 | 4.6655 | -0.33325 | -0.5332 |
| **3** | -1.333 | 10.230775 | 3.999 | -0.33325 | -0.3999 |
| **4** | -0.6665 | 9.5976 | 2.99925 | 0 | -0.633175 |
