# Supplementary material for: Effects of vehicle gap changes on fuel economy and emission performance of the traffic flow in the ACC strategy
Source: PLoS One. 2018 Jul 12;13(7):e0200110. doi: 10.1371/journal.pone.0200110 (PMC6042720; doi:10.1371/journal.pone.0200110)
Supplement: S2 Table — (DOC) [file pone.0200110.s002.doc]

**S2 Table. Partial measured CF data (δ=2s**)

| **t** | **a_1_(m/s^2^)** | **d_21_(m)** | **v_1_(m/s)** | **Δv_21_(m/s)** | **Δxc_21_(m/s)** |
| --- | --- | --- | --- | --- | --- |
| **1** | -0.6665 | 5.098725 | 4.33225 | 0.33325 | 0.299925 |
| **2** | -0.6665 | 4.86545 | 3.66575 | -0.6665 | -0.233275 |
| **3** | -1.333 | 4.33225 | 2.666 | -0.6665 | -0.766475 |
| **1** | -0.6665 | 4.698825 | 4.99875 | 0.33325 | 1.099725 |
| **2** | -0.6665 | 4.33225 | 4.33225 | -0.6665 | 0.06665 |
| **3** | -1.333 | 3.699075 | 3.3325 | -0.33325 | -0.99975 |
| **1** | -0.6665 | 14.096475 | 4.33225 | -1.66625 | -3.8657 |
| **2** | -0.6665 | 12.096975 | 3.66575 | -1.66625 | -3.93235 |
| **3** | -0.6665 | 9.9975 | 2.99925 | -1.9995 | -4.098975 |
| **1** | -0.6665 | 5.8652 | 4.33225 | 0 | -0.46655 |
| **2** | -0.6665 | 5.431975 | 3.66575 | -0.33325 | -0.699825 |
| **3** | -0.6665 | 4.565525 | 2.99925 | -0.99975 | -1.299675 |
| **1** | -0.6665 | 6.19845 | 4.99875 | -0.6665 | -0.166625 |
| **2** | -0.6665 | 5.93185 | 4.33225 | -0.6665 | -0.499875 |
| **3** | -0.6665 | 5.53195 | 3.66575 | -0.6665 | -0.6665 |
| **4** | -0.6665 | 4.99875 | 2.99925 | -0.6665 | -0.9331 |
| **1** | -0.6665 | 6.7983 | 4.33225 | -0.6665 | -1.1997 |
| **2** | -0.6665 | 5.93185 | 3.66575 | -0.6665 | -1.432975 |
| **3** | -0.6665 | 5.165375 | 2.99925 | -0.33325 | -1.632925 |
| **1** | -1.333 | 3.632425 | 3.3325 | -0.33325 | -0.566525 |
| **2** | 0 | 3.4658 | 2.666 | 0 | -0.499875 |
| **3** | -0.6665 | 3.165875 | 2.33275 | -0.33325 | -0.46655 |
| **4** | -0.6665 | 2.699325 | 1.66625 | -0.33325 | -0.766475 |
| **1** | -1.333 | 6.59835 | 5.332 | -0.6665 | -1.0664 |
| **2** | -0.6665 | 6.2651 | 4.33225 | 0 | -0.6665 |
| **3** | 0 | 5.7319 | 3.999 | -0.6665 | -0.86645 |
| **1** | -1.333 | 5.032075 | 5.332 | -0.99975 | -3.565775 |
| **2** | -0.6665 | 3.79905 | 4.33225 | -0.6665 | -2.965925 |
| **3** | -0.6665 | 3.232525 | 3.66575 | -0.99975 | -1.79955 |
| **1** | -1.333 | 4.59885 | 4.6655 | -0.6665 | -1.099725 |
| **2** | -0.6665 | 3.999 | 3.66575 | 0 | -1.26635 |
| **3** | 0 | 3.765725 | 3.3325 | -0.33325 | -0.833125 |
| **4** | -0.6665 | 2.9326 | 2.99925 | -1.333 | -1.0664 |
| **1** | -0.6665 | 4.232275 | 4.99875 | -0.33325 | 0.033325 |
| **2** | -1.333 | 3.5991 | 3.999 | -0.6665 | -0.833125 |
| **3** | -0.6665 | 2.99925 | 2.99925 | -0.6665 | -1.233025 |
| **1** | -0.6665 | 11.5971 | 4.99875 | -0.6665 | -1.8662 |
| **2** | 0 | 10.364075 | 4.6655 | -0.99975 | -2.19945 |
| **3** | -0.6665 | 9.097725 | 4.33225 | -1.66625 | -2.499375 |
| **1** | 0 | 6.365075 | 4.6655 | 0.33325 | 0.3999 |
| **2** | -1.333 | 5.8652 | 3.999 | 0 | -0.3999 |
| **3** | 0 | 4.965425 | 3.3325 | -0.33325 | -1.39965 |
| **4** | -0.6665 | 4.06565 | 2.99925 | -1.333 | -1.79955 |
| **1** | -0.6665 | 8.33125 | 3.66575 | -0.6665 | -0.1333 |
| **2** | -0.6665 | 7.4648 | 2.99925 | -0.6665 | -1.033075 |
| **3** | -0.6665 | 6.19845 | 2.33275 | -0.6665 | -2.1328 |
| **1** | -0.6665 | 9.1977 | 4.99875 | -1.333 | -2.499375 |
| **2** | -1.333 | 7.698075 | 3.999 | -0.99975 | -2.99925 |
| **3** | -1.333 | 6.33175 | 2.666 | -0.6665 | -2.86595 |
| **1** | -0.6665 | 2.1328 | 2.99925 | 0.6665 | -0.33325 |
| **2** | 0 | 2.232775 | 2.666 | 0.33325 | 0.033325 |
| **3** | -1.333 | 2.032825 | 1.9995 | -0.33325 | -0.099975 |
| **1** | 0 | 5.0654 | 3.999 | -0.6665 | -1.233025 |
| **2** | -1.333 | 4.098975 | 3.3325 | -0.99975 | -1.699575 |
| **3** | -0.6665 | 3.26585 | 2.33275 | -0.6665 | -1.79955 |
| **4** | -0.6665 | 2.46605 | 1.66625 | -0.6665 | -1.632925 |
