# Supplementary material for: Effects of vehicle gap changes on fuel economy and emission performance of the traffic flow in the ACC strategy
Source: PLoS One. 2018 Jul 12;13(7):e0200110. doi: 10.1371/journal.pone.0200110 (PMC6042720; doi:10.1371/journal.pone.0200110)
Supplement: S3 Table — (DOC) [file pone.0200110.s003.doc]

**S3 Table. Partial measured CF data (δ=3s**)

| **t** | **a1(m/s2)** | **d21(m)** | **v1(m/s)** | **Δv21(m/s)** | **Δxc21(m/s)** |
| --- | --- | --- | --- | --- | --- |
| **1** | -0.6665 | 4.86545 | 3.66575 | -0.6665 | 0.06665 |
| **2** | -1.333 | 4.33225 | 2.666 | -0.6665 | -0.766475 |
| **1** | -0.6665 | 4.33225 | 4.33225 | -0.6665 | 0.73315 |
| **2** | -1.333 | 3.699075 | 3.3325 | -0.33325 | -0.566525 |
| **1** | -0.6665 | 12.096975 | 3.66575 | -1.66625 | -5.8652 |
| **2** | -0.6665 | 9.9975 | 2.99925 | -1.9995 | -6.031825 |
| **1** | -0.6665 | 5.431975 | 3.66575 | -0.33325 | -0.899775 |
| **2** | -0.6665 | 4.565525 | 2.99925 | -0.99975 | -1.566275 |
| **1** | -0.6665 | 5.93185 | 4.33225 | -0.6665 | -0.433225 |
| **2** | -0.6665 | 5.53195 | 3.66575 | -0.6665 | -0.899775 |
| **3** | -0.6665 | 4.99875 | 2.99925 | -0.6665 | -1.1997 |
| **1** | -0.6665 | 5.93185 | 3.66575 | -0.6665 | -2.06615 |
| **2** | -0.6665 | 5.165375 | 2.99925 | -0.33325 | -2.19945 |
| **1** | 0 | 3.4658 | 2.666 | 0 | -0.73315 |
| **2** | -0.6665 | 3.165875 | 2.33275 | -0.33325 | -0.7998 |
| **3** | -0.6665 | 2.699325 | 1.66625 | -0.33325 | -0.9331 |
| **1** | -0.6665 | 6.2651 | 4.33225 | 0 | -1.39965 |
| **2** | 0 | 5.7319 | 3.999 | -0.6665 | -1.1997 |
| **1** | -0.6665 | 3.999 | 3.66575 | 0 | -1.699575 |
| **2** | 0 | 3.765725 | 3.3325 | -0.33325 | -1.499625 |
| **3** | -0.6665 | 2.9326 | 2.99925 | -1.333 | -1.66625 |
| **1** | -1.333 | 5.8652 | 3.999 | 0 | -0.099975 |
| **2** | 0 | 4.965425 | 3.3325 | -0.33325 | -1.299675 |
| **3** | -0.6665 | 4.06565 | 2.99925 | -1.333 | -2.299425 |
| **1** | -1.333 | 4.098975 | 3.3325 | -0.99975 | -2.19945 |
| **2** | -0.6665 | 3.26585 | 2.33275 | -0.6665 | -2.5327 |
| **3** | -0.6665 | 2.46605 | 1.66625 | -0.6665 | -2.59935 |
