# Supplementary material for: Effects of vehicle gap changes on fuel economy and emission performance of the traffic flow in the ACC strategy
Source: PLoS One. 2018 Jul 12;13(7):e0200110. doi: 10.1371/journal.pone.0200110 (PMC6042720; doi:10.1371/journal.pone.0200110)
Supplement: S4 Table — (DOC) [file pone.0200110.s004.doc]

**S4 Table. The related coefficients in Eq. (6)**

|  | **Fuel** | **CO** | **HC** | **NOx** |
| --- | --- | --- | --- | --- |
| $K_{0,0}^{e}$ | -0.679439 | 0.887447 | -0.728042 | -1.067682 |
| $K_{0,1}^{e}$ | 0.135273 | 0.148841 | 0.012211 | 0.254363 |
| $K_{0,2}^{e}$ | 0.015946 | 0.030550 | 0.023371 | 0.008866 |
| $K_{0,3}^{e}$ | -0.001189 | -0.001348 | -0.00009324 | -0.000951 |
| $K_{1,0}^{e}$ | 0.029665 | 0.070994 | 0.024950 | 0.046423 |
| $K_{2,0}^{e}$ | -0.000276 | -0.000786 | -0.000205 | -0.000173 |
| $K_{3,0}^{e}$ | 0.000001487 | 0.00000462 | 0.00000195 | 0.00000057 |
| $K_{1,1}^{e}$ | 0.004808 | 0.003870 | 0.010145 | 0.015482 |
| $K_{1,2}^{e}$ | -0.00002054 | 0.00009323 | -0.000103 | -0.000131 |
| $K_{1,3}^{e}$ | 5.540929E-8 | -0.00000071 | 0.00000062 | 0.00000033 |
| $K_{2,1}^{e}$ | 0.000083329 | -0.000926 | -0.000549 | 0.002876 |
| $K_{2,2}^{e}$ | 0.000000937 | 0.00004918 | 0.00003759 | -0.0000587 |
| $K_{2,3}^{e}$ | -2.479644E-8 | -0.00000031 | -0.00000021 | 0.00000024 |
| $K_{3,1}^{e}$ | -0.00006132 | 0.00004614 | -0.000113 | -0.000321 |
| $K_{3,2}^{e}$ | 0.000000304 | -0.00000141 | 0.00000331 | 0.00000194 |
| $K_{3,3}^{e}$ | -4.467234E-9 | 8.172401E-9 | -1.73937E-8 | -1.25741E-8 |
